# Cardiac Tamponade Caused by a Needle in the Liver Presenting as a STEMI Mimic

**DOI:** 10.1016/j.jaccas.2025.105932

**Published:** 2025-12-17

**Authors:** Yen Wing Ng, Hamish I.J. Elliott, Philip A. Curry, Nigel B. Jamieson, John G. Dreisbach, Angie Ghattas, Andrew P. Apps

**Affiliations:** a Department of Interventional Cardiology, Golden Jubilee National Hospital, Clydebank, United Kingdom; b Department of Cardiothoracic Surgery, Golden Jubilee National Hospital, Clydebank, United Kingdom; c Department of Hepatopancreatobiliary Surgery, Glasgow Royal Infirmary, Glasgow, United Kingdom; d Department of Radiology, Golden Jubilee National Hospital, Clydebank, United Kingdom

**Keywords:** computed tomography, pericardial effusion, tamponade

## Abstract

**Background:**

We report a case of hemorrhagic pericardial effusion with tamponade, secondary to myocardial injury by a hypodermic needle lost during narcotics injection some months prior.

**Case Summary:**

First presentation was with chest pain and global ST segment elevation following earlier cocaine usage. Urgent coronary angiography was performed to rule out acute coronary artery occlusion. A needle-like foreign body was seen abutting the right ventricle free wall, and the invasive pressure waveform suggested ventricular interdependence. Transthoracic echocardiogram revealed large-volume pericardial effusion which was drained immediately. The needle was ultimately removed during laparotomy and partial hepatectomy.

**Discussion:**

This case is unique and demonstrates how all available clinical information was collated to diagnose and treat life-threatening extra-coronary pathology with a rare etiology, in a patient whose history was unreliable.

**Take-Home Message:**

The case highlights the importance of maintaining a broad differential diagnosis during emergency angiography in the presence of ST-segment elevation if no coronary occlusion is found.

## Graphical Abstract

**Figure:**
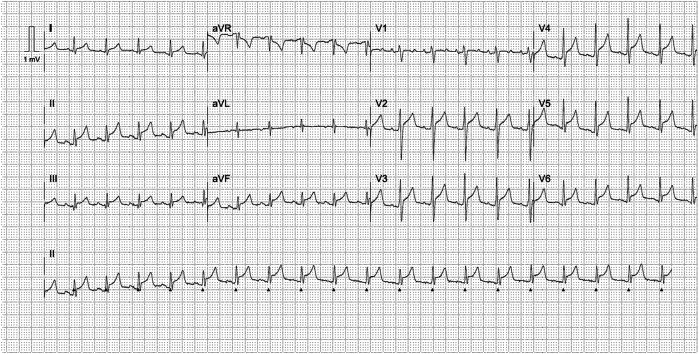

## History of Presentation

**Figure 1:**
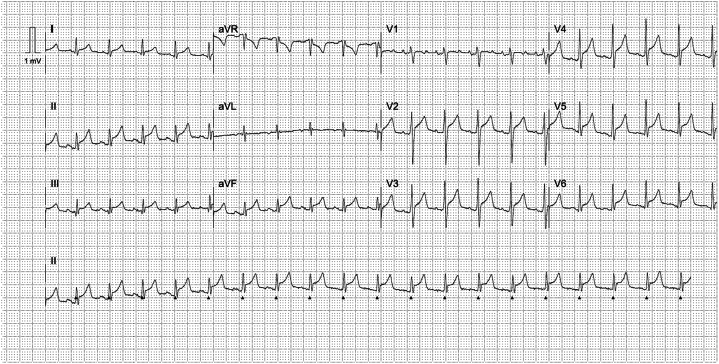
Initial Electrocardiogram Initial electrocardiogram demonstrating sinus tachycardia with global concave pattern of st-segment elevation.

A 44-year-old heavy smoker from the West of Scotland presented with sharp nonradiating central chest pain, not positional in nature, with acute onset after cocaine usage hours earlier. In the field on first medical attendance, the patient was diaphoretic, agitated, cool to touch, and in an early state of clinical shock with blood pressure 79/58 mm Hg and heart rate 120 beats/min. Temperature was 36.8 °C, and respiratory rate was 20 breaths/min. Cardiac examination revealed no rub or murmur; the jugular venous pressure was elevated; lung fields were clear. Electrocardiogram showed a global pattern of ST-segment elevation (Figure 1).

**Take-Home Messages**

• The case highlights the importance of maintaining a broad differential diagnosis during emergency presentations in the context of ST elevation on the electrocardiogram; here to diagnose and treat life-threatening extra-coronary pathology.
• Repetitive sensitive history-taking is crucial when key information is not immediately forthcoming, potentially due to its sensitive nature.
• Multidisciplinary working is key to deliver good care in complex cases.

## Past Medical History

The only medical history available at presentation was of previous intravenous drug use, and untreated Hepatitis C infection. On more direct questioning during his subsequent medical stay, a history of needle loss during drug use became apparent, initially denied at presentation. The details offered varied and included needle loss during femoral injection, loss during a direct attempt to inject in the epigastric area toward the heart, and being stabbed with a needle during an assault. Despite inconsistency, the reported timing was consistently reported as 5 months prior to presentation.

## Differential Diagnosis

The differential diagnosis included acute coronary artery occlusion, due to vasospasm or dissection following cocaine usage, or more classical coronary artery disease with plaque rupture. Myopericarditis, due to Hepatitis C infection or chronic cocaine use, was also a possibility given a concave global ST-segment elevation pattern on the electrocardiogram. Progressively deteriorating hemodynamics was in keeping with cardiogenic shock complicating one of these diagnoses.

## Investigations

**Figure 2:**
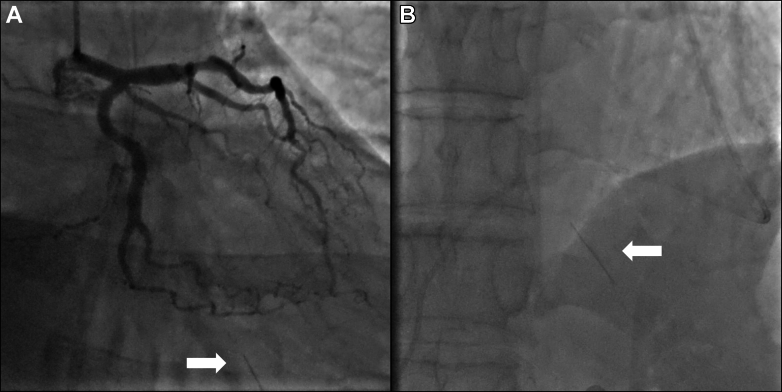
Emergency Invasive Coronary Angiography and Pericardiocentesis Invasive coronary angiogram with right anterior oblique caudal view (A) showing a needle-like opacity (white arrow) which moved with respiration located inferior to the heart. The object was also evident on fluoroscopy (white arrow) during emergency pericardiocentesis (B).

**Figure 3:**
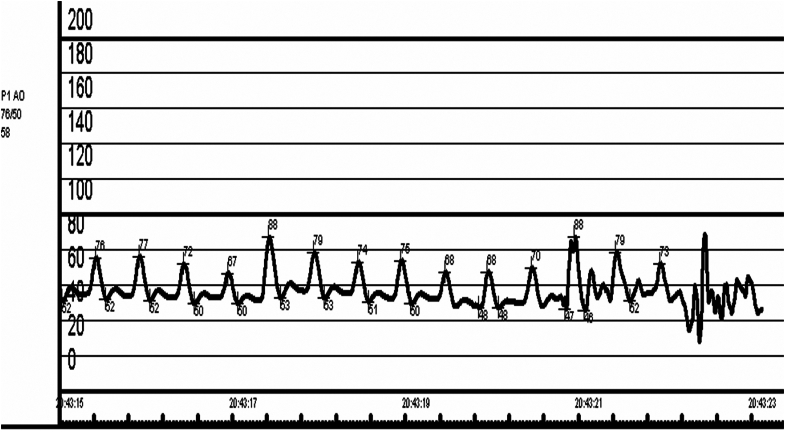
Invasive Aortic Pressure Tracing Invasive aortic pressure tracing demonstrating significant respiratory variation, with the systolic pressure dropping from 88 mm Hg to 67 mm Hg during inspiration. This was in keeping with ventricular interdependence and pericardial pathology.

**Figure 4:**
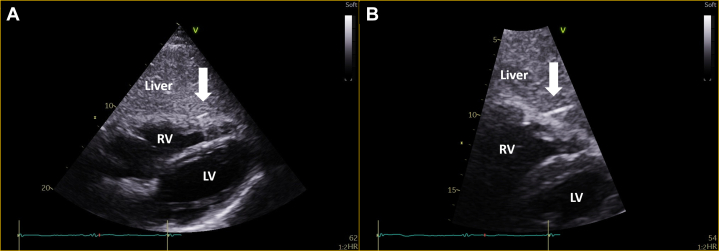
Transthoracic Echocardiography Transthoracic echocardiogram in subcostal windows (A) and (B) demonstrating the foreign body (white arrow) embedded in the liver, in close proximity to the right ventricle free wall. LV = left ventricle; RV = right ventricle.

**Figure 5:**
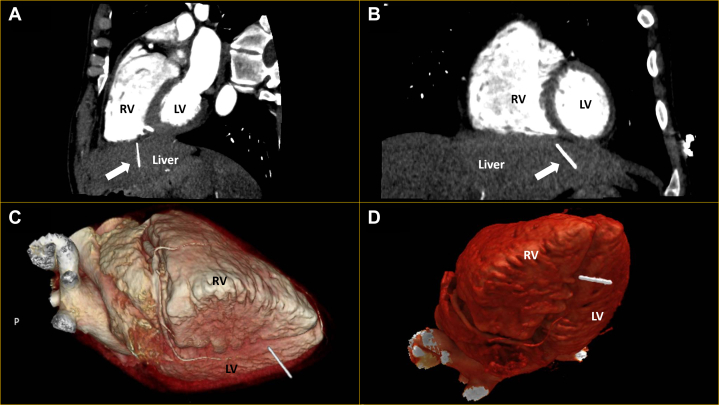
Cardiac-Gated Computed Tomography With 3D Reconstructions Cardiac-gated computed tomography in oblique sagittal (A) and coronal (B) views, as well as 3D reconstructions (C and D) demonstrate the 25-mm needle (white arrow) in the left liver lobe oriented toward the heart and 60-mm deep from the skin surface. LV = left ventricle; RV = right ventricle.

With ST-segment elevation after cocaine use, as well as classical risk factors for coronary artery disease in an area known for having the highest incidence of coronary artery disease in Europe,^1^ an emergency angiogram was undertaken to rule out acute coronary occlusion. Normal TIMI flow grades of III were found in all epicardial coronary arteries, with moderate plaque disease in the proximal left anterior descending vessel. Caudal angiographic views demonstrated a needle-like foreign body located craniocaudally adjacent to the inferior right ventricle myocardium, moving with the cardiac cycle (Figure 2, Videos 1 and 2). Furthermore, exaggerated swings in blood pressure (∼18 mm Hg) were noted throughout the respiratory cycle, suggesting ventricular interdependence and significant pericardial pathology (Figure 3). Subsequent transthoracic echocardiography confirmed large-volume pericardial effusion; emergency pericardiocentesis via a xiphisternal approach drained 500 mL of frank blood with normalization of hemodynamics. A highly echogenic structure was seen within the pericardium, abutting the right ventricle free wall (Figure 4, Videos 3 and 4). Cardiac-gated computed tomography confirmed a 25-mm needle-like object positioned in the parenchyma of the left liver lobe oriented craniocaudally toward the posterior interventricular septum, approximately 2 mm below the mid-inferior right-ventricle myocardium, with the cranial end of the needle tip straddling the pericardium, particularly in diastole. The needle was about 60 mm deep from the skin surface (Figure 5).

## Management

**Figure 6:**
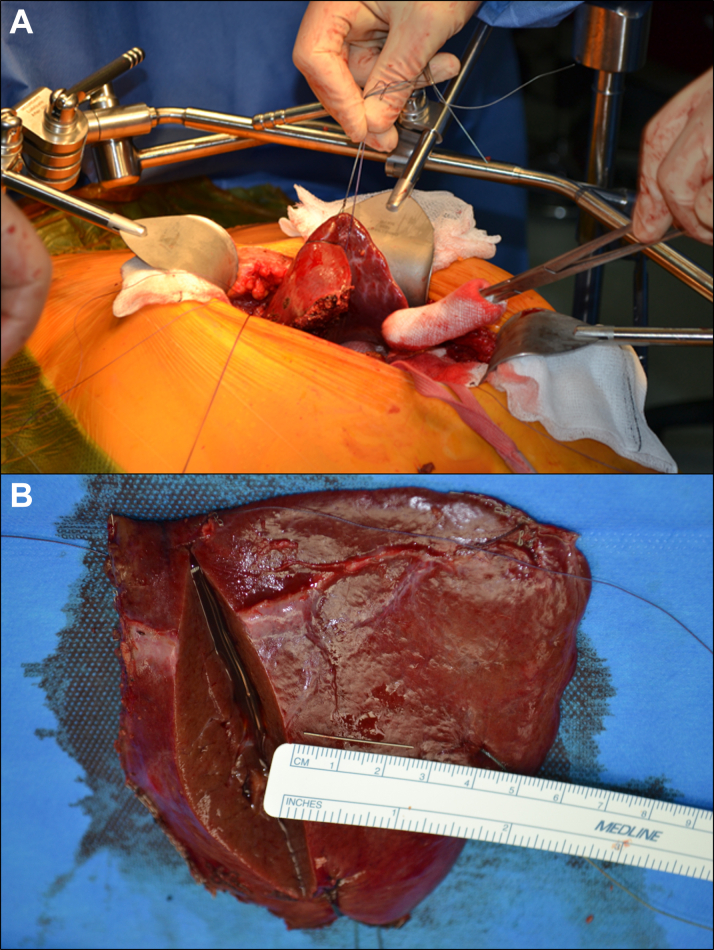
Partial Hepatectomy Partial hepatectomy was performed given that the needle was not easily extractable despite localization with ultrasound during surgery (A). The needle is subsequently shown once removed from the resected segment (B).

The patient ultimately stabilized, and the drain was removed. The cardiac multidisciplinary team felt that the needle was causal to his presentation and should be removed surgically to reduce the risk of direct penetrative myocardial injury. As the body of this object was deep within vascular structures within the left liver lobe, this was planned as an open hepatobiliary case with cardiothoracic cover in case of mediastinal bleeding mandating emergency thoracotomy. Surgery was performed via an upper midline incision under general anesthesia. Once the liver was exposed, direct ultrasound guidance identified the needle to be more than 3 cm deep and not easily extractable directly. Therefore, a left lateral segment partial hepatectomy was performed successfully (Figure 6). The patient had an uneventful recovery and was discharged 4 days after the surgery.

## Outcome and Follow-Up

The patient had an uneventful recovery and was discharged from the intensive care unit 1 day after surgery and discharged home 3 days later. Cardiology and hepatobiliary follow-up is planned in his local hospital.

## Discussion

This case of a life-threatening presentation of cardiac tamponade is unique for a number of reasons and provides points for reflection. The underlying etiology here is of a retained foreign body caused by a trauma remote to the presentation (6 months). The needle presumably during this time migrated to a position within the pericardial space causing bleeding; however, the initial entry site of the needle is unclear. The case highlights the importance of maintaining a broad differential diagnosis during emergency angiography in the presence of ST-segment elevation if no coronary occlusion is found. Here, all clinical information, including extra-cardiac findings during angiography, characteristics of the invasive arterial pressure waveform, and direct questioning of the patient during the procedure, prompted echocardiography and life-saving pericardiocentesis. Admittedly, concave ST-segment elevation, chest pain, and pericardial collection is relatively common, but the cause in our patient is certainly unique. Mechanistically, it is unclear how recent cocaine usage finally prompted pericardial bleeding, but it is likely that the associated adrenergic stimulation finally prompted bleeding and tamponade physiology. Cocaine usage may, however, be a red herring—a potentially unhelpful one, invoking the differential of coronary vasospasm or dissection.

The patient's liver function remained normal during admission. In a Hepatitis C carrier, there may be a long-term cost of partial hepatectomy, as is seen during partial hepatectomy for malignancy in the context of Hepatitis C.^2^ However, with the needle situated deep in the left lobe of the liver and at an unusual angle, it was challenging to extract directly without causing extensive injury. Therefore, partial hepatectomy was performed, requiring the left lobe of the liver to be fully mobilized, made harder by the need to minimize direct contact in order to prevent needle-stick injury.

## Conclusions

This case demonstrates the value of retaining a broad index of clinical suspicion to ultimately make a diagnosis in patients with atypical presentations to primary percutaneous coronary intervention services. Here, a highly unusual extra-coronary diagnosis was made and resolved in both the acute phase of tamponade and chronically by hepatobiliary surgery. The case also demonstrates the value in persistently and sensitively seeking information from the patient when history is not forthcoming due to its potentially sensitive nature and the value of multidisciplinary working to ensure safe care.

## Funding Support and Author Disclosures

The authors have reported that they have no relationships relevant to the contents of this paper to disclose.

## Appendix

Video 1 Invasive Coronary Angiogram With Right Anterior Oblique Caudal View Showing a Needle-Like Opacity Which Moved With Respiration Located Inferior to the Heart

Video 2 Invasive Coronary Angiogram With Right Anterior Oblique Caudal View Showing a Needle-Like Opacity Which Moved With Respiration Located Inferior to the Heart

Video 3 Echocardiogram With Subcostal Views Showing the Needle Position Relative to the Heart

Video 4 Echocardiogram With Subcostal Views Showing the Needle Position Relative to the Heart
